# Arrival of Paleo-Indians to the Southern Cone of South America: New Clues from Mitogenomes

**DOI:** 10.1371/journal.pone.0051311

**Published:** 2012-12-11

**Authors:** Michelle de Saint Pierre, Francesca Gandini, Ugo A. Perego, Martin Bodner, Alberto Gómez-Carballa, Daniel Corach, Norman Angerhofer, Scott R. Woodward, Ornella Semino, Antonio Salas, Walther Parson, Mauricio Moraga, Alessandro Achilli, Antonio Torroni, Anna Olivieri

**Affiliations:** 1 Instituto de Ecología y Biodiversidad, Departamento de Ecología, Facultad de Ciencias, Universidad de Chile, Ñuñoa, Santiago, Chile; 2 Programa de Genética Humana, Instituto de Ciencias Biomédicas, Facultad de Medicina, Universidad de Chile, Independencia, Santiago, Chile; 3 Dipartimento di Biologia e Biotecnologie, Università di Pavia, Pavia, Italy; 4 Sorenson Molecular Genealogy Foundation, Salt Lake City, Utah, United States of America; 5 Institute of Legal Medicine, Innsbruck Medical University, Innsbruck, Austria; 6 Unidade de Xenética, Departamento de Anatomía Patolóxica e Ciencias Forenses, and Instituto de Ciencias Forenses, Facultade de Medicina, Universidad de Santiago de Compostela, Santiago de Compostela, Galicia, Spain; 7 Servicio de Huellas Digitales Genéticas, Facultad de Farmacia y Bioquímica, Universidad de Buenos Aires, Buenos Aires, Argentina; 8 AncestryDNA, Provo, Utah, United States of America; 9 Eberly College of Science, Penn State University, University Park, Pennsylvania, United States of America; 10 Departamento de Antropología, Facultad de Ciencias Sociales, Universidad de Chile, Ñuñoa, Santiago, Chile; 11 Dipartimento di Biologia Cellulare e Ambientale, Università di Perugia, Perugia, Italy; Democritus University of Thrace, Greece

## Abstract

With analyses of entire mitogenomes, studies of Native American mitochondrial DNA (mtDNA) variation have entered the final phase of phylogenetic refinement: the dissection of the founding haplogroups into clades that arose in America during and after human arrival and spread. Ages and geographic distributions of these clades could provide novel clues on the colonization processes of the different regions of the double continent. As for the Southern Cone of South America, this approach has recently allowed the identification of two local clades (D1g and D1j) whose age estimates agree with the dating of the earliest archaeological sites in South America, indicating that Paleo-Indians might have reached that region from Beringia in less than 2000 years. In this study, we sequenced 46 mitogenomes belonging to two additional clades, termed B2i2 (former B2l) and C1b13, which were recently identified on the basis of mtDNA control-region data and whose geographical distributions appear to be restricted to Chile and Argentina. We confirm that their mutational motifs most likely arose in the Southern Cone region. However, the age estimate for B2i2 and C1b13 (11–13,000 years) appears to be younger than those of other local clades. The difference could reflect the different evolutionary origins of the distinct South American-specific sub-haplogroups, with some being already present, at different times and locations, at the very front of the expansion wave in South America, and others originating later *in situ*, when the tribalization process had already begun. A delayed origin of a few thousand years in one of the locally derived populations, possibly in the central part of Chile, would have limited the geographical and ethnic diffusion of B2i2 and explain the present-day occurrence that appears to be mainly confined to the Tehuelche and Araucanian-speaking groups.

## Introduction

The study of the first peopling of the Americas represents one of the first and most significant examples of fruitful interaction between archeology, linguistics and genetics [1]. Archeologists and anthropologists were the first to hypothesize an initial entry of Native American ancestors from Siberia across Beringia, a land bridge made accessible by a substantial lowering of the sea-level toward the end of the last Ice Age [2]. In recent decades, genetics has provided novel data and techniques to shed light on America’s first colonizers, particularly regarding the timing of their arrival and the routes they took (for a review see [2]–[4] and references therein). The combined "archeogenetic" approach has provided further clues on the colonization process, with novel data provided by one discipline reinforcing or dismissing the scenarios proposed by the other. Archeology has recently witnessed the downfall of the “Clovis-first” theory – envisioning an entry time, not prior to 13 thousand years ago (kya), which is in agreement with the dating of the Clovis culture in North America – and the staggering discovery of pre-Clovis sites in Monteverde (Chile) [5]–[6] and Texas [7], both dated as early as 15.5–14.5 kya [8]. Major genetic contributions have come from mitochondrial DNA (mtDNA) studies, mainly carried out in modern populations, but also with a non-negligible and steadily increasing input from ancient human remains [9]–[12]. Increasing data support the scenario that the ancestors of Paleo-Indians settled in Beringia before the Last Glacial Maximum (LGM), which may have later forced them into distinct enclaves when climatic conditions worsened. This initial and fragmented Beringian gene pool, despite the probably narrow time window of about 5 ky [13] was dynamic, with novel mtDNA mutations arising *in situ* and a continuous reshaping not only due to drift, but also to bidirectional gene flow with northeastern Asia [14]–[15]. This shaped the mutational motifs of Native American mitochondrial lineages and created lineage composition differences in the distinct enclaves. Starting from about 15–18 kya, a rapid southward expansion took Paleo-Indians from Beringia all the way to the extreme southern tip of South America, covering a latitude gap of more than 100° (from about 65° North to 54° South) and a distance of more than 15,000 km, possibly in a time span of less than 2,000 years [16]–[17]. These initial migrations likely occurred following two entry ways: the Pacific coastal route, probably playing the major role in the peopling of the double continent, and the ice-free corridor passage between the Laurentide and Cordilleran ice sheets, that also had a significant impact, at least on the colonization of northern North America [18]–[23].

In very recent years, in parallel with the refinement of the worldwide mtDNA phylogeny (see [24]), the resolution of Native American-specific haplogroups has improved. Due to the sequencing of entire mitogenomes, the overall number of recognized maternal founding lineages has gone from just four - initially named A, B, C and D [25]–[27] - to a current count of 16 [16], [22]. Among these, eight haplogroups – A2, B2, C1b, C1c, C1d (including C1d1), D1 and D4h3a – are pan-American, as they are distributed across the double continent [14], [19], [21], [28]–[29], while the remaining are less frequent and generally show a distribution restricted to North America (A2a, A2b, C4c, D2a, D3, D4e1, X2a and X2g) [16], [19], [21], [23], [29]–[34].

It is widely accepted that, when all Native American lineages – not only the Asian and Beringian founders, but also those that originated *in situ* during the colonization process – are analyzed at the level of mitogenomes over their entire (past and present) distribution range, more comprehensive conclusions on migration and timing will become feasible [17]. Therefore, current and future studies should also focus on geographically restricted, sometimes rare, mtDNA clades, which can contribute additional details to the overall and/or local picture of the peopling of the Americas. Examples come from some very recent studies: Hooshiar Kashani et al. [23] focused on C4c, a rare founding haplogroup possibly marking an ice-free corridor entry; Perego et al. [35] defined an ancient lower Central American branch, termed A2af, within the pan-American A2; whereas Gómez-Carballa et al. [36] began to identify extremely young local clades such as the Venezuelan B2j and B2k. As for the southern part of South America, Bodner et al. [17] identified two novel sub-clades within the pan-American haplogroup D1, named D1g and D1j, which are restricted to populations of the Southern Cone and most likely marked the first human arrival in the region about 15 kya.

The South American Southern Cone is of extreme interest for genetic investigations because: (i) it is the most distant area from the Beringian source, thus it was likely reached during the final phases of the peopling of the Americas, (ii) it houses one of the most ancient archeological sites of the entire continent (Monteverde, ∼14.5 ky) [2], and (iii) it is crossed in length by the Andes, a potential major barrier to latitudinal migratory events. Great effort has been employed to assess the mtDNA variation in populations from the Southern Cone (Chile and Argentina) [37]–[43]. However, analyses have generally focused solely on the sequence information of a portion of the mtDNA control region (often only the hypervariable segment I - HVS-I). The recent work of Bodner et al. [17] was the first attempt to analyze the Southern Cone mtDNA variation at the level of complete sequences by focusing on two specific clades within the pan-American founder haplogroup D1.

In a very recent study, the mtDNA control-region sequence variation of 300 native people from Chile and Argentina was analyzed and two additional subsets of mtDNAs were identified [43]. In particular, one subset harbored a transition at nucleotide position (np) 470 in the context of haplogroup B2, while the other group, in addition to the mutational motif for haplogroup C1b, shared the transition at np 258. These two new potential Southern Cone-specific sub-haplogroups were provisionally named B2l and C1b13 [43]. The aim of the present study is to further investigate the origin of these clades by employing the information contained in the whole mtDNA molecule. To accomplish this task, 25 putative B2l and 21 putative C1b13 mitogenomes were sequenced. Ages and phylogeographic data of the two haplogroups were evaluated, also in comparison with those of the previously described Southern Cone-specific sub-haplogroups D1g and D1j [17].

## Results

### Phylogeny and Age Estimates of the Two Novel Southern Cone mtDNA Haplogroups

**Figure 1:**
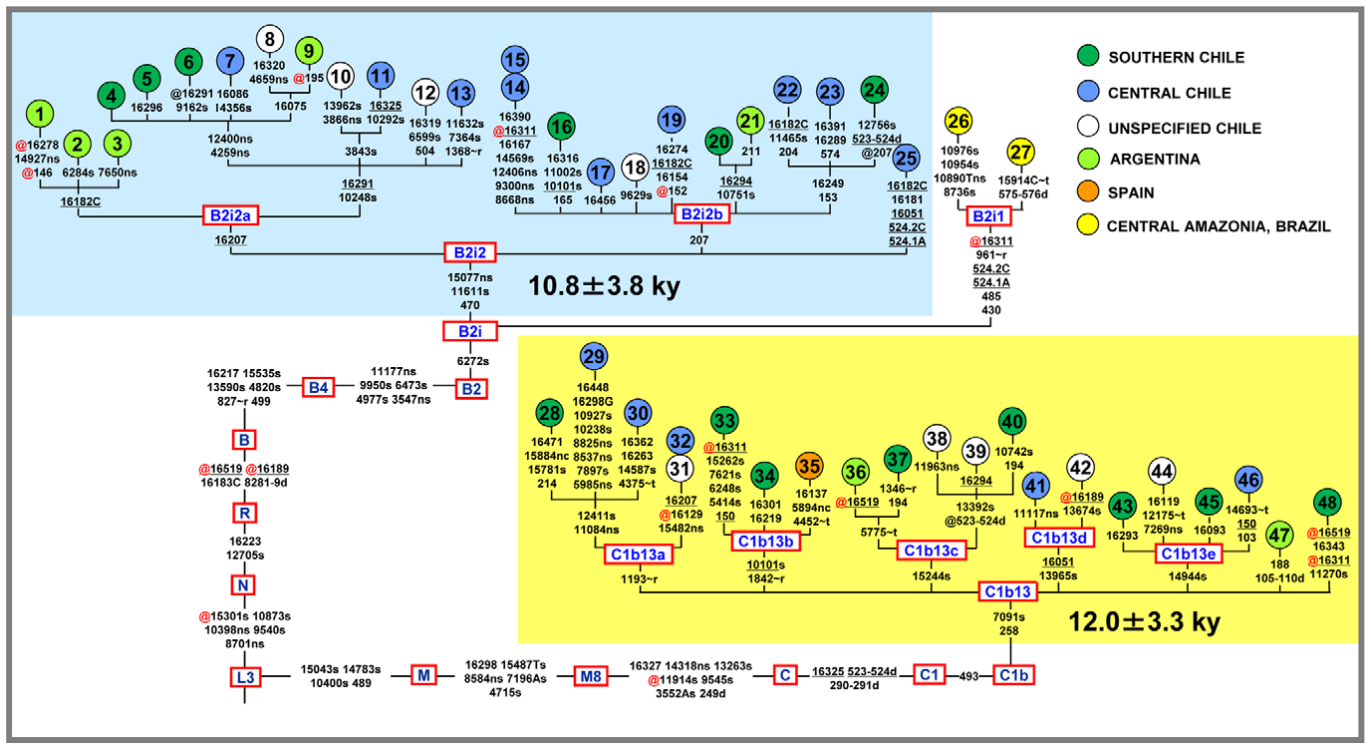
Detailed maximum parsimony tree of 46 novel complete Native American mtDNA sequences belonging to the novel haplogroups B2i2 and C1b13. These are the first completely sequenced mitogenomes for both B2i2 and C1b13. This tree also includes two previously published sequences of Kayapó individuals from Brazil [19] classified as belonging to sub-clade B2i1. Mutations relative to the L3 node are shown on the branches; they are transitions unless a base is explicitly indicated. The prefix @ indicates reversions while suffixes indicate: transversions (to A, G, C, or T), indels (.1, d), gene locus (∼r, rRNA; ∼t, tRNA), synonymous or non-synonymous changes (s or ns), and non-coding sites outside the control region (nc). The mutations marked by a red @ are reverted only relative to the Revised Sapiens Reference Sequence (RSRS) [24], all other mutations are relative to both rCRS [75] and RSRS. Recurrent mutations within the phylogeny are underlined. The variation in number of cytosines around nps 309 and 16193 was not included in the tree. Additional information regarding each mtDNA is available in Table 1. Coalescence times shown for B2i2 and C1b13 are Maximum-Likelihood (ML) estimates, and have been obtained by including all sequence changes (except 16182C, 16183C, and at np 16519) from the respective root according to Soares et al. [78].

The phylogenetic relationships of the 46 selected mitochondrial genomes are illustrated in Figure 1. Additional information concerning the geographic and ethnic origin of each mtDNA is provided in Table 1.

**Table 1:** List of mtDNA haplogroup B2i and C1b13 complete sequences included in Figure 1.

ID #^a^	Sample ID	Haplogroup	Geographic Origin	Ethnic Affiliation	GenBank ID	Reference
**1**	ARN083^b^	B2i2a	Rio Negro, Argentina	Argentinian (unknown)	JX413011	**This study**
**2**	Mco32	B2i2a	Neuquén, Argentina	Mapuche	JX413012	**This study**
**3**	Mco34	B2i2a	Neuquén, Argentina	Mapuche	JX413013	**This study**
**4**	D04	B2i2a	Detif, Chiloe Island, Chile	Chilean (rural)	JX413014	**This study**
**5**	CA007	B2i2a	Carelmapu, Chiloe Island, Chile	Chilean (rural)	JX413015	**This study**
**6**	CA012	B2i2a	Carelmapu, Chiloe Island, Chile	Chilean (rural)	JX413016	**This study**
**7**	XL060	B2i2a	San Felipe, Aconcagua, Chile	Chilean (urban)	JX413017	**This study**
**8**	686289	B2i2a	Chile	Chilean (unknown)	JX413018	**This study**
**9**	ARN086^b^	B2i2a	Rio Negro, Argentina	Argentinian (unknown)	JX413019	**This study**
**10**	686571	B2i2a	Chile	Chilean (unknown)	JX413020	**This study**
**11**	XL119	B2i2a	San Felipe, Aconcagua, Chile	Chilean (urban)	JX413021	**This study**
**12**	933177	B2i2a	Chile	Chilean (unknown)	JX413022	**This study**
**13**	XL144	B2i2a	Llay-Llay, Aconcagua, Chile	Chilean (urban)	JX413023	**This study**
**14**	XL058	B2i2b	Los Andes, Aconcagua, Chile	Chilean (urban)	JX413024	**This study**
**15**	XL030	B2i2b	San Felipe, Aconcagua, Chile	Chilean (urban)	JX413025	**This study**
**16**	CA028	B2i2b	Carelmapu, Chiloe Island, Chile	Chilean (rural)	JX413026	**This study**
**17**	XL050	B2i2b	Valparaiso, Chile	Chilean (urban)	JX413027	**This study**
**18**	686246	B2i2b	Chile	Chilean (unknown)	JX413028	**This study**
**19**	XL156	B2i2b	San Felipe, Aconcagua, Chile	Chilean (urban)	JX413029	**This study**
**20**	CA046	B2i2b	Carelmapu, Chiloe Island, Chile	Chilean (rural)	JX413030	**This study**
**21**	ARN109^b^	B2i2b	Rio Negro, Argentina	Argentinian (unknown)	JX413031	**This study**
**22**	XL061	B2i2b	Los Andes, Aconcagua, Chile	Chilean (urban)	JX413032	**This study**
**23**	XL012	B2i2b	Llay-Llay, Aconcagua, Chile	Chilean (urban)	JX413033	**This study**
**24**	H05^c^	B2i2b	San Juan de la Costa, Chile	Huilliche	JX413034	**This study**
**25**	MSP	B2i2	Santiago, Chile	Chilean (urban)	JX413035	**This study**
**26**	KBK39	B2i1	Amazonia, Brazil	Kayapó	EU095217	[**19**]
**27**	KKT01	B2i1	Amazonia, Brazil	Kayapó	EU095218	[**19**]
**28**	H19^c^	C1b13a	San Juan de la Costa, Chile	Huilliche	JX413036	**This study**
**29**	XL193	C1b13a	Los Andes, Aconcagua, Chile	Chilean (urban)	JX413037	**This study**
**30**	XL036	C1b13a	San Felipe, Aconcagua, Chile	Chilean (urban)	JX413038	**This study**
**31**	686331	C1b13a	Chile	Chilean (urban)	JX413039	**This study**
**32**	XL028	C1b13a	Los Andes, Aconcagua, Chile	Chilean (urban)	JX413040	**This study**
**33**	H08^c^	C1b13b	San Juan de la Costa, Chile	Huilliche	JX413041	**This study**
**34**	QUE009	C1b13b	Quetalmahue, Chiloe Island, Chile	Chilean (rural)	JX413042	**This study**
**35**	686537	C1b13b	Spain^d^	/	JX413043	**This study**
**36**	Mco13	C1b13c	Neuquén, Argentina	Mapuche	JX413044	**This study**
**37**	L016	C1b13c	Laitec, Chiloe Island, Chile	Chilean (rural)	JX413045	**This study**
**38**	686478	C1b13c	Chile	Chilean (unknown)	JX413046	**This study**
**39**	686156	C1b13c	Chile	Chilean (unknown)	JX413047	**This study**
**40**	T38^c^	C1b13c	Trapa Trapa, Chile	Pehuenche	JX413048	**This study**
**41**	XL003	C1b13d	San Felipe, Aconcagua, Chile	Chilean (urban)	JX413049	**This study**
**42**	686285	C1b13d	Chile	Chilean (unknown)	JX413050	**This study**
**43**	QUE0012	C1b13e	Quetalmahue, Chiloe Island, Chile	Chilean (rural)	JX413051	**This study**
**44**	686497	C1b13e	Chile	Chilean (unknown)	JX413052	**This study**
**45**	L006	C1b13e	Laitec, Chiloe Island, Chile	Chilean (rural)	JX413053	**This study**
**46**	XL187	C1b13e	Los Andes, Aconcagua, Chile	Chilean (urban)	JX413054	**This study**
**47**	SA18^e^	C1b13	Salta, Argentina	Kolla	JX413055	**This study**
**48**	CA045	C1b13	Carelmapu, Chiloe Island, Chile	Chilean (rural)	JX413056	**This study**

a ID numbers correspond to the numbers in Figure 1.

b The control-region sequence of these mtDNAs were previously published by Bobillo et al. [42].

c Partial control-region sequences of these mtDNAs were previously published by de Saint Pierre et al. [43].

d The maternal grandmother of subject n. 35 was born in Talagante, Chile.

e The HVS-I sequence of this mtDNA was previously published by Álvarez-Iglesias et al. [41].

Note that the 25 B2l mtDNAs harboring the transition at np 470 were reclassified in this study as members of haplogroup B2i2. This change in nomenclature was required because the transition at np 6272, a distinguishing coding-region mutation present in all our mtDNAs, is shared with a clade, previously identified by Fagundes et al. [19], which encompasses the complete genomes of two Kayapó individuals from Brazilian Amazonia (Figure 1). The “Kayapó clade” was recently named B2i1 (Phylotree, Build 15 [44]). Therefore, the haplogroup nomenclature of our “B2l” mitogenomes was consistently updated to B2i2, a novel sub-haplogroup that is defined by the mutational motif 470-11611-15077. All B2i2 haplotypes, with the exception of one sequence (#25), cluster into two sub-clades, termed B2i2a and B2i2b, both defined by a single control-region transition at np 16207 and np 207, respectively.

Haplogroup C1b13 encompasses 21 mitogenomes and radiates from the root of C1b with the mutational motif 258–7091. This haplogroup exhibits ample diversity with at least five major basal branches (C1b13a–C1b13e) (Figure 1), each defined by at least one coding-region mutation.

The Maximum Likelihood (ML) divergences for haplogroups B2i2 and C1b13 are very similar (4.07±0.70 and 4.50±0.60, respectively) (Table 2) and correspond to coalescence times of 10.8±3.8 and 12.0±3.3 ky, respectively (Figure 1). These ages were overall confirmed when the average distances of the haplotypes from the root of haplogroups B2i2 and C1b13 (ρ-statistics) were computed (Table 2) (rho and sigma values of 5.04±1.03 and 4.24±0.64), corresponding to an age of 13.5±5.6 ky for B2i2 and 11.3±3.5 ky for C1b13.

**Table 2:** Molecular divergence and age estimates (Maximum Likelihood and rho statistics) for Southern Cone-specific mtDNA haplogroups.

Haplogroup	All nucleotide substitutions								
	*N* a	ML^b^	S.E.	Age (ky)^c^	95% CI (ky)	ρ	σ	Age (ky)^c^	95% CI (ky)
**B2i**	27	7.0	1.3	19.3	{12.2; 26.6}	7.9	1.9	21.7	{11.3; 32.5}
**> B2i2**	25	4.1	0.7	10.8	{7.1; 14.6}	5.0	1.0	13.5	{8.0; 19.2}
**C1b13**	21	4.5	0.6	12.0	{8.8; 15.3}	4.2	0.6	11.3	{7.85; 14.8}
**D1g** d	26	6.7	0.8	18.3	{15.9; 20.7}	7.2	1.0	19.7	{16.7; 22.7}
**D1j** d	17	5.2	1.0	13.9	{11.0; 16.8}	5.5	1.7	14.9	{10.2; 19.6}

a Number of mtDNA sequences.

b The maximum likelihood molecular divergence.

c Using the corrected molecular clock proposed by Soares et al. [78].

d Haplogroups D1g and D1j are included for comparison. Data are from Bodner et al. [17].

Age estimates for haplogroup B2i as a whole could also be potentially informative. However, clade B2i1 is represented by only two sequences, thus the overall time estimates for B2i are for the moment rather loose: 19.3±7.2 ky (ML) and 21.7±10.6 ky (ρ-statistics) (Table 2).

To evaluate a possible role of selection on the sequence evolution of haplogroups B2i2 and C1b13, the numbers of synonymous and non-synonymous substitutions in the 13 protein coding genes of the mitogenomes were investigated using the neutrality tests described by Elson et al. [45] and Ruiz-Pesini et al. [46]. Resulting neutrality indices obtained by testing the two haplogroups, both individually (B2i2: I/T = 4.1, Ni = 0.25, P>0.05; C1b13: I/T = 0.5, Ni = 2, P>0.05) and together (I/T = 1.4, Ni = 0.7, P>0.05), were not significant.

### Phylogeography of Haplogroups B2i2 and C1b13

All mitogenomes sequenced in this study derived from Chile and Argentina, with the exception of one C1b13 mtDNA sample from Spain (sample #35 in Figure 1), whose maternal origin could be traced back to Chile (Table 1). To further evaluate the geographical distribution of the two haplogroups, we extended our search of B2i2 and C1b13 control-region mutational motifs to published datasets from both Native American groups and national populations of North, Central and South America. By searching the Sorenson Molecular Genealogy Foundation [47] control-region mtDNA database, the European DNA Profiling Group Mitochondrial Population Database (EMPOP) [48], and a database of more than 7,000 Native American mtDNA control-region sequences (in house database, A. Salas), we confirmed that all subjects bearing the B2i2 and C1b13 mutational motifs shared the same origin in the southern part of South America. The results of this survey provide further support to the scenario [43] that, similar to haplogroups D1g and D1j [17], both B2i2 and C1b13 are virtually restricted to the Southern Cone of South America (Table 3).

**Table 3:** Percentage frequencies of Southern Cone-specific mtDNA haplogroups in local Native American groups and national populations estimated from control-region data.

Country, Population or Region	n	Haplogroups				Reference
		B2i2^a^	C1b13^b^	D1g	D1j	
***Chile:***						
Atacameño	28	0	3.6	0	3.6	[43]
Aymara	39	0	0	2.6	2.6	[43]
Huilliche	58	25.9	15.5	37.9	0	[43]
Kawésqar	13	0	7.7	0	0	[80]
Mapuche	34	ND	23.5	26.5	0	[39]
Mapuche	19	26.3	26.3	15.8	0	[43]
Pehuenche	42	26.2	28.6	38.1	0	[43]
Pehuenche	24	ND	33.3	25.0	0	[39]
Yámana	36	0	11.1	33.3	0	[39], [43]
Chileans	729	14.5	19.1	13.9	0.4	[47]
***Argentina:***						
Catamarca Province	25	ND	4.0	20.0	28.0	[14]
Colla	60	0	1.7	0	1.7	[41]
Mapuche	39	ND	17.9	15.4	10.5	[37]
Mapuche	90	38.9	11.1	20.0	2.2	[43]; Sala A & Corach D, unpubl. data
Pilagá	38	ND	ND	0	2.6	[40]
Wichí	99	ND	ND	0	2.0	[40]
Mocovi	5	0	0	0	20.0	[14]
Tehuelche	57	14.0	21.1	29.8	0	[43]; Sala A & Corach D, unpubl. data
Argentinians	497	4.6	5.8	7.2	2.8	[42], [47]; Bobillo MC in [48]; Sala A & Corach D, unpubl. data
Argentinians	179	ND	1.1	1.7	3.9	Vullo C in [48]
Argentinians	384	ND	0.3	1.3	5.5	[81]
Argentinians (Center)	102	ND	ND	2.9	6.9	[82]
Fuegian-Patagonians (ancient DNA)	24	0	ND	8.3	0	[64]
Fuegian-Patagonians (ancient DNA)	60	0	ND	ND	ND	[63]
***Neighboring countries:***						
Bolivians	187	0	0	0	0	[47]
Brazilians	1362	0	0	0.2	0.1	[47]
Paraguayans	32	0	0	0	0	[47]
Peruvians	2005	0	0.1	0	0	[47]
Uruguayans	116	0	0	0.9	0	[47]

a The frequency of B2i2 is often not determined (ND) because its diagnostic control-region mutation at np 470 is outside HVS-I and was generally not covered by mtDNA studies on Native Americans.

b The frequency of C1b13 is often not determined (ND) because its diagnostic control-region mutation at np 258 is outside HVS-I and was generally not covered by mtDNA studies on Native Americans.

## Discussion

The first peopling of the Americas has fascinated scholars from different disciplines for centuries. A major milestone was reached in the 1920s with the discovery of the so-called Clovis culture when Aleš Hrdlička published his theories of a Siberian origin of Native American populations, coming into North America by crossing the current Bering Strait [49]. However, only in recent decades did archeological, linguistic and genetic evidence [1], [25]–[27], [38], [50]–[56] begin to provide scenarios congruent enough to answer the long-standing questions in Native American studies – when and from where did the first Americans arrive, and what migratory routes did they follow? The mitochondrial genome, despite its small size, played a pivotal role. MtDNA studies in the early 1990s identified the major founding maternal lineages of the first settlers [25], [50], [57]. Following this initial approach and with the advent of complete mitochondrial sequencing, an impressive increase in the level of phylogenetic resolution was obtained, bringing the total number of identified founding mtDNA sequences from Beringia/Asia to 16, including both widespread (pan-American) and geographically-restricted haplogroups. In more recent years, studies of Native American mtDNA variation entered the final phase of the phylogenetic refinement process: the molecular dissection of the founding haplogroups into sub-clades of younger age and more restricted geographic and population distribution [17], [33], [35]. A paradigmatic example of the power of this approach in a different continental context (Western Eurasia) is represented by haplogroup H. The pivotal work by Achilli and collaborators [58] identified the first 15 clades within H, which in just eight years grew to 87 in number [24], with countless internal branches. This fine dissection revealed informative spatial patterns attributable to a number of distinct dispersal and migratory events [59]–[62].

The present study is a further example of the “magnifying glass” approach applied to Native American-specific haplogroups. The dissection of the major pan-American haplogroups, which began in 2008 [19], [33], is further extended by analyzing two clades, termed B2i2 and C1b13, whose geographical distributions appear to be restricted to Chile and Argentina. This feature supports the scenario that the mutational motifs characterizing these sub-haplogroups arose in South America, probably in the Southern Cone region [43].

While both sub-haplogroups B2i2 and C1b13 are restricted to the Southern Cone, their spatial distributions are not identical. Haplogroup B2i2 is found at high frequencies in the Mapuche of Chile (26.3%) and Argentina (38.9%), Pehuenche (26.2%), Huilliche (25.9%) and Tehuelche (14.0%) (Table 3), all populations living in the central-southern part of Chile and Argentina and belonging to the Araucanian language family, except the Tehuelche, who belong to the Chon language family. B2i2 mtDNAs appear to be instead absent in more northern (Atacameño and Aymara) and southern (Kawésqar and Yámana) native groups. The absence of B2i2 mtDNAs in Tierra del Fuego/southern Patagonian populations is also supported by the overall absence of B2 mtDNAs in pre-Columbian human remains of that area [63]–[64]. In contrast, the geographic and ethnic distribution of C1b13 appears to be wider both towards the North and the South. It encompasses not only Native American groups of the central-southern part of the Southern Cone, but also the Kawésqar and Yámana of the extreme South and the Atacameño of northern Chile [43].

From currently available data, the geographic distributions of both B2i2 and C1b13 appear to be more restricted than those reported for the two southern Cone-specific haplogroups identified by Bodner et al. [17], especially relative to D1j, which is observed possibly even in the ancient Tainos of the Dominican Republic [17], [65]. Taken together, as already evidenced by de Saint Pierre et al. [43], haplogroups B2i2, C1b13, D1g and D1j, despite their rare occurrences within the overall Native American context, can locally reach extremely high frequencies, even up to 80–90% as observed in the Huilliche and Pehuenche of Chile and the Mapuche of Argentina (Table 3). Their largely overlapping distributions strongly support the scenario that they might have been characterized, at least in part, by parallel evolutionary histories.

Most likely, the molecular ancestors of the four founding haplotypes that arrived in the Southern Cone were carried by the pioneer human groups following the southward route along the Pacific coast, as proposed by Bodner et al. [17] for haplogroups D1g and D1j. This is in agreement with the observation that the eastern populations of South America exhibit lower levels of heterozygosity for different genetic systems, and suggests an initial colonization of the western part of South America and a subsequent peopling of the eastern area by western subgroups [51], [66]–[69]. The recent study by Reich et al. [70] adds further support to the Pacific Coast as a facilitator for migrations during the initial settlement of the double continent. However, the four Southern Cone-specific sub-haplogroups, with this study now each characterized by well-defined mutational motifs, could have originated at different times and different locations during the process of human expansion along the Pacific Coast. If the mutational motif arose at the very front of the expansion wave and just prior to its arrival in what is now Chile, the age estimate of the corresponding haplogroup would tend to correspond with that of the human colonization of the Southern Cone. In such a scenario, it is also likely that the sub-haplogroup would have been present in all, or at least many (considering genetic drift) of the derived populations along the Pacific coast of the Southern Cone – and in the continental inland taking into account the following trans-Andean migrations [17]. Alternatively, the mutational motif could have originated later, in one of the (probably numerous) derived population groups that arose locally along the trail of the colonization wave across the Pacific coastal areas of the Southern Cone. In this latter scenario, the age estimate of the sub-haplogroup would be younger than the time of the first arrival in the area and its spatial distribution more restricted, encompassing only a portion of the Southern Cone region.

From the dispersal patterns and ages of the four known Southern Cone-specific clades, B2i2, C1b13, D1g and D1j, it is likely that both envisioned scenarios apply to the process of human colonization of the Southern Cone. Indeed, the four sub-haplogroups do not always show overlapping coalescence ages. For sub-haplogroups B2i2 and C1b13, we obtained ML ages that are rather similar to each other (10.8±3.8 and 12.0±3.3 ky, respectively; Table 2), but younger than those of D1g and D1j, whose ML ages were estimated at 18.3±2.4 and 13.9±2.9 ky, respectively, by Bodner et al. [17] (Table 2). The difference, especially the one between the youngest (B2i2) and the oldest (D1g) might be due to a sampling bias similar to the one that initially affected the age estimate of C1d [22], but could also reflect truly different evolutionary origins of the sub-haplogroups, with D1g being already present in the pioneer settlers who first colonized the Pacific coastal regions of the Southern Cone (i.e. the first scenario described above), whereas B2i2 could have originated later, after the initial colonization of the extreme South, when the tribalization process had already begun, from an intermediate mtDNA haplotype placed between the B2i and B2i2 nodes (Figure 1; Table 2) already present in the pioneering wave (i.e. the second scenario described above). A delayed origin of a few thousand years in one of the locally derived populations, possibly in the central part of what is now Chile, would have limited the geographical and ethnic diffusion of B2i2 and explain the present-day occurrence that appears to be mainly confined to the Tehuelche and the Araucanian-speaking groups living in the more central area of the Southern Cone.

As mentioned above, the mutational link at np 6272 between the sister clades B2i1 and B2i2 was discovered only after entire mitochondrial genomes of Native American origin were sequenced. To date we have a very limited number of mitogenomes from South America. However, we know that two distinct B2i1 sequences are present in the Kayapò of Brazilian Amazonia. To obtain additional information concerning the geographic distribution of this clade, we searched the Sorenson Molecular Genealogy Foundation [47] control-region mtDNA database for the control-region mutational motif of B2i1 (146-152-195-247-315.1C-430-485-499-524.1A-524.2C-16129-16183C-16187-16217-16223-16230-16278 relative to the RSRS, which corresponds to the motif 73-263-315.1C-430-485-499-524.1A-524.2C-16183C-16189-16217-16311-16519 relative to the rCRS). We identified only two additional mtDNAs, one from Brazil and one from northern Uruguay (both bearing the B2 control-region haplotype plus the B2i diagnostic transitions at np 430 and 485), thus preliminarily suggesting a geographic distribution of B2i1 limited to the northern and eastern part of South America.

This observation is preliminary, but provides some clues on the possible origin of B2i as a whole. It raises in fact the possibility that the transition at np 6272, which is the distinguishing mutation of B2i, occurred on a B2 mtDNA either prior to the arrival of the first human settlers in South America or soon afterwards in a northern area of South America. The preliminary age estimates for B2i as a whole (Table 2) are compatible with this possibility. Such a scenario could also imply that the early B2i mtDNAs not only moved from northern South America along the Pacific, giving rise to the full mutational motif of sub-haplogroup B2i2 only later in the Southern Cone, but they might have also expanded from the same northern area of South America, possibly after an incubation period [17], towards the eastern part of South America, generating later what we now call haplogroup B2i1. In other words, the identification of the mutational link between haplogroups B2i2 and B2i1, the first apparently restricted to the Southern Cone and the second possibly restricted to North East, could be interpreted as supporting the early population split into coastal and continental population groups previously proposed by several anthropological and genetic studies [51], [66]–[69], [71]–[74].

In conclusion, our data support the previously proposed scenario of a rapid colonization of South America through the Pacific coastal route and provide first insights into additional, more complex migration events. This North to South expansion was marked by the occurrence of novel sub-haplogroups, such as B2i and D1g, which probably arose, at different times and locations, at the front of the colonization wave. The defining mutation of B2i possibly occurred prior to or soon afterward the entry of Paleo-Indians in South America and might have been involved in an early split of the first settlers in the northern part of South America. Sub-haplogroups such as B2i, whose clade composition can only be defined by a systematic survey of entire mitogenomes derived from Native Americans, might be the ideal tools to trace and date the earliest human steps in South America. Haplogroup D1g probably arose at the front of the colonization wave but later in the population group that had already taken the Pacific route [17], perhaps just prior to its entry in the northern regions of Chile, thus later spreading along the entire south-western coastal line. Finally, the mutational motifs of other sub-haplogroups, such as B2i2 and C1b13, might have been fully completed even more recently, in specific populations of the Pacific regions of the Southern Cone, when the process of linguistic differentiation and tribalization had already begun. These mtDNA clades which differentiated *in situ* within a few thousand years after human arrival could represent excellent markers to investigate the trans-Andean movements [17] which, after the initial expansion along the Pacific coastal regions, probably led to the colonization of the entire Southern Cone of South America.

## Materials and Methods

### Sample Selection, Ethics Statement and Analysis of mtDNA Sequence Variation

Candidate B2i2 (former B2l) and C1b13 mtDNAs were identified and selected by screening the mtDNA control region of subjects from native and general populations of Chile and Argentina [43] and by searching the Sorenson Molecular Genealogy Foundation (SMGF) control-region mtDNA database (∼80,000 subjects [47]), the European DNA Profiling Group Mitochondrial Population Database (EMPOP) [48], and a database of more than 7,000 Native American mtDNA control-region sequences (in house database, A. Salas). To include the widest range of original variation of the two sub-haplogroups, we preferred mtDNAs from subjects of the general (rural and urban) populations of Chile and Argentina rather than subjects from indigenous groups (Table 1), which are often, especially for mtDNA, prone to genetic drift and founder events. Therefore only four of the subjects previously analyzed by de Saint Pierre et al. [43] were included in this study. As for B2i2, potential members were identified based on the presence of the B2 control-region motif 146-152-195-247-315.1C-499-16129-16183C-16187-16217-16223-16230-16278-16311 relative to the Revised Sapiens Reference Sequence (RSRS, [24]), which corresponds to the motif 73-263-315.1C-499-16183C-16189-16217-16519 relative to rCRS [75], plus the B2i2 diagnostic transition at np 470 [43]. MtDNAs with the C1b control-region motif 146-152-195-247-249d-290d-291d-315.1C-489-493-523d-524d-16129-16187-16189-16230-16278-16298-16311-16325-16327-16519 relative to RSRS (73-249d-263-290d-291d-315.1C-489-493-16223-16298-16325-16327 relative to rCRS) plus the C1b13 diagnostic transition at np 258 [43] were considered possible members of C1b13. A total of 46 candidate mtDNAs were then completely sequenced. Of these, 25 (20 from Chile and five from Argentina) and 21 (18 from Chile, two from Argentina and one from Spain, whose maternal grandmother was born in Chile) harbored the B2i2 and C1b13 motifs, respectively. The geographic and ethnic affiliations of the 46 mtDNAs are listed in table 1, together with the GenBank accession number of the corresponding sequence. For all subjects, appropriate written informed consent was obtained, and the research was approved by the Ethics Committee for Clinical Experimentation of the University of Pavia, Board minutes of the 5th of October, 2010. Sequencing of entire mitochondrial genomes was performed as previously described [76]. In brief, a set of 11 overlapping PCR fragments covering the entire mtDNA genome was produced and sequenced by standard chain termination sequencing with 32 nested oligonucleotides. Complete sequences were aligned to the RSRS [24], assembled, and compared using Sequencher 4.9 (Gene Codes). Phylogeny construction was performed by hand following a maximum parsimony approach.

### Age Estimates

To obtain the maximum likelihood (ML) molecular divergences of haplogroups B2i2 and C1b13, we used PAML 4.4 [77], assuming the HKY85 mutation model (with indels ignored, as usual) with gamma-distributed rates (approximated by a discrete distribution with 32 categories) and three partitions: HVS-I (positions 16051 to 16400), HVS-II (positions 68 to 263), and the remainder. The ML estimates were then compared with those directly obtained from the averaged distance (*ρ*) of the haplotypes of a clade to the respective root haplotype accompanied by a heuristic estimate of the standard error (σ) calculated from an estimate of the genealogy. This calculation was performed on entire mtDNA haplotypes (excluding variants 16182C, 16183C, and 16519). Mutational distances were converted into years using the corrected molecular clock proposed by Soares et al. [78].

To evaluate a possible role of selection on haplogroup age estimates, neutrality tests by Elson et al. [45] and Ruiz-Pesini et al. [46] were performed using the mtPhyl program [79]. Synonymous (s) and non-synonymous (ns) substitutions in mitogenomes were stratified into two classes: one including substitutions shared by at least two mtDNAs, the other encompassing private substitutions occurring at the tips of individual branches. The significance of the differences in ns:s ratios between two classes was determined on the basis of the Fisher’s exact test (two tails).
